# Goettingen Minipigs (GMP): Comparison of Two Different Models for Inducing Diabetes

**DOI:** 10.1186/1758-5996-4-7

**Published:** 2012-03-05

**Authors:** Armin Strauss, Vasily Moskalenko, Christian Tiurbe, Irina Chodnevskaja, Stephan Timm, Verena A Wiegering, Christoph-Thomas Germer, Karin Ulrichs

**Affiliations:** 1 Department of General, Visceral, Vascular and Pedatric Surgery (Department of Surgery I)University Hospital of Wuerzburg, Germany; 2 Maltese Hospital and St. Franziskus Hospital, Flensburg, Germany; 3 Experimental Transplantation Immunology, Department of General, Visceral, Vascular and Paediatric Surgery (Surgical Clinic I), University Hospital of Wuerzburg, Germany; 4 Department of Paediatrics, University Hospital of Wuerzburg, Germany

**Keywords:** diabetes, pig or swine, real-time glucose monitoring, intravenous glucose tolerance test, total pancreatectomy, streptozotocin

## Abstract

**Purpose:**

Preclinical experiments on large animals are indispensable for evaluating the effectiveness of diabetes therapies. Miniature swine are well suited for such studies due to their physiological and pathophysiological responses.

**Methods:**

We compare two methods for inducing diabetes in Goettingen minipigs (GMP), in five with the beta cell toxin streptozotocin (STZ) and in five other GMP by total pancreatectomy (PE). Glucose homeostasis was assessed with the intravenous glucose-tolerance test (IVGTT) and continual monitoring of interstitial glucose levels. At conclusion of the observation period, the pancreata were examined histologically. Three non-diabetic GMP served as control group.

**Results:**

The IVGTT revealed markedly diabetic profiles in both GMP groups. STZ-GMP were found to harbor residual C-peptides and scattered insulin-positive cells in the pancreas. PE-GMP survived the total pancreatectomy only with intensive postoperative care.

**Conclusions:**

Although both methods reliably induced diabetes in GMP, the PE-GMP clearly had more health problems and required a greater expenditure of time and resources. The PE-GMP model, however, was better at eliminating endogenous insulin and C-peptide than the STZ-GMP model.

## Introduction

Neither the physiology of glucose metabolism [1,2] nor the pathophysiologies of types 1 and 2 diabetes mellitus [3-8] are fully understood. Among the promising methods for treating type 1 diabetes is the transplantation of isolated microencapsulated islets of Langerhans [9,10]; metabolic surgery is one of the recently introduced treatment options for type 2 diabetes [11,12]. Many open questions regarding experimental diabetes treatment modalities have been answered in studies using small animal diabetes models. Such studies, for example, have answered fundamental questions regarding the appropriate transplantation site for islets cells [13,14], the function of islets cell transplants *in vivo *[15,16], the recipient immune response to islets cell transplants [17], and regarding postoperative glucose homeostasis in the field of diabetes metabolism research [18]. As valuable as such studies are, however, questions regarding the feasibility of such concepts under preclinical conditions can only be answered in large animal models of diabetes.

Although non-human primates are ideally suited for such studies due to their genetic and physiological proximity to humans, ethical considerations and the personnel required and the high associated costs have limited primate studies to a very minimum. For training in the course of transplantation the minipig is a serious alternative because of its physiological resemblance to humans, but also due to its size, ease of handling, and the much lower costs entailed. Because spontaneous diabetes is unknown in swine, the diabetes must be induced. The present study compares two different diabetes models in Goettingen minipigs (GMP), one model inducing diabetes chemically with the beta cell toxin streptozotocin (STZ), the other inducing diabetes surgically by total pancreatectomy (PE).

Both variants have already been described in the literature [19-22]. The results with STZ are largely uniform: the severity of the induced diabetes depends on the STZ dosage. The results with PE are less consistent. Postoperative survival of the animals has been reported as less than 10 days, which would disqualify PE as a diabetes-induction method. But several reports describe diabetic swine and canines that have undergone PE followed by transplantation of islets of Langerhans and remained free of postoperative complications during a long term survival of several months [23,24]. The present study evaluates and compares the advantages and disadvantages of each of the two models for diabetes induction in GMP. The findings will be used to establish an optimal large animal model for research into specific questions of diabetes therapy.

## Materials and methods

### Animals

Ten 11 to 17-month-old female GMP weighing 20-35 kg were studied. The GMP were housed individually under standardized conditions (19-23°C; 40-70% relative humidity; 12:12 hour day/night cycle); they were fed once daily with a standard swine feed (Altromin; Lage, Germany); water was provided *ad libitum*. All experiments were approved in advance by the government of Lower Franconia.

### Diabetes induction with STZ

In 5 GMP, 150 mg/kg body weight (bw) of the beta cell toxin STZ (Sigma-Chemie, Munich, Germany) were infused via a central venous catheter over 10 min (buffered solution: 1 g STZ in 10 mL sodium citrate buffer freshly prepared). To avoid hypoglycemia due to insulin release by the destroyed beta cells, 200 mL 5% glucose solution were given over one hour after STZ application. The observation time in this group was 3-4 months.

### Diabetes induction with *total *pancreatectomy

Five GMP underwent total pancreatectomy under general anesthesia. After transverse upper abdominal laparotomy, the pancreas tail was carefully detached from the surrounding tissue. Small vessels originating in the spleen and the V. portae were ligated and cut. The spleen was always left intact. Next the corpus and caput were detached from the surrounding tissue while sparing the pancreaticoduodenal vessels and the vessels supplying the duodenum. Lastly the pancreatic duct was exposed, double ligated and cut. The abdomen was closed and once the anesthetic wore off the animal was returned to its pen where it received intensive medical care. Over the next 5 days the PE-GMP were given analgesia with 1 g Novalgin^® ^(Aventis Pharma; Frankfurt am Main, Germany) and 3 × 50 mg Tramal^® ^(Ratiopharm; Ulm, Germany) daily. Prophylactic antimicrobial therapy was also administered with Tazobac^® ^EF4 g/0.5 g (Wyeth Pharma GmbH; Muenster, Germany). When feeding was begun on the 6th to 8th postoperative day, the GMP received an exogenous pancreas enzyme (Creon^® ^25,000 IE/Tag) (Solvay Arzneimittel; Hanover, Germany) at every feeding. The observation time for this group was 28 days.

### Real time glucose monitoring

The CGMS^® ^(Medtronic MiniMed; Northridge, USA) continuous glucose monitoring system was used as described by us previously [25].

### Blood sugar monitoring

All blood sugar readings were done on capillary blood from the ears. The first drop was discarded; the second was placed on a test strip of the Ascensia Contour^® ^blood glucose monitoring system (Bayer Health Care; Mishawaka, USA).

### Intravenous glucose tolerance test and hyperglycemic clamp

The intravenous glucose tolerance test (IVGTT) was performed between the 20th and 28th days after diabetes induction. The values of the diabetic GMP were compared with those of the normoglycemic control GMP. Feeding and insulin treatment were discontinued 12-16 h before IVGTT was performed. At time point t = 0 0.5 g/kg bw glucose was injected. At time points t = -20 min; -10; 0; +1; +3; +5; +7; +10; +20; +30; +40; and +60 min blood samples were taken and the serum insulin and C-peptide levels measured. At the same time blood glucose levels were determined. For the subsequent hyperglycemic clamp in normoglycemic animals, 0.6 g glucose pro kg bw were given at t = +60 min to raise the blood glucose level above 500 mg/dL, followed by continuous glucose infusion (2 g/kg bw/hour) to maintain blood glucose levels at over 500 mg/dL till t = +100 min, then the infusion was stopped. At t = +90 min 67 mg arginine/kg bw was administered. Blood samples for monitoring insulin and C-peptide as well as blood glucose values were taken at time points t = +60 min, +61, +63, +65, +67, +70, +80, +90, +91, +93, +95, +97, +100 and +110 min. In diabetic GMP there was no renewed application of glucose solution or continuous glucose infusion. Arginine was given at t = +60 min. Blood glucose levels were measured and blood samples taken at t = +60 min, +61, +63, +65, +67, +70, +80 and +90 min.

### C-peptide RIA

C-peptide was determined in GMP serum samples using the RIA-Kit (PCP-22K, Linco Research; Missouri, USA). The resulting radioactivity was measured with a Gamma counter (Berthold; Bad Wildbad, Germany) in *counts per minute (*cpm). C-peptide in ng/mL was calculated using a standard curve.

### Insulin ELISA

Serum insulin levels were measured with a commercial ELISA-Kit (Biosource; Nivelles, Belgium). The samples were analyzed at two wavelengths (450 nm and 490 nm) against a reference filter (650 nm) with the aid of an ELISA reader (Thermo Max Microplate Reader, MWG Biotech; Ebersberg, Germany). Insulin levels were measured on a standard curve and shown in μU/mL.

### Histology

At conclusion of the observation time, the pancreata of the STZ-GMP were removed and 1 cm^3 ^tissue blocks from the head, corpus and tail were cryoconserved in liquid nitrogen. Sections from the different sites of the pancreas were prepared from these blocks. The sections were stained with standard hematoxylin-eosin (H&E) and for standard immunohistochemistry with an antiinsulin antibody (Guinea pig-anti-swine antiserum; Dako; Hamburg, Germany).

### Statistics

All data are given as mean ± standard deviation (SD).

## Results

### Glucose metabolism in normoglycemic non-diabetic control GMP

Continuous interstitial glucose monitoring and glucose tolerance tests were performed in 3 adult, 11 to 17-month-old female non-diabetic GMP weighing 20 to 35 kg.

#### Real-time glucose monitoring in control GMP

**Figure 1:**
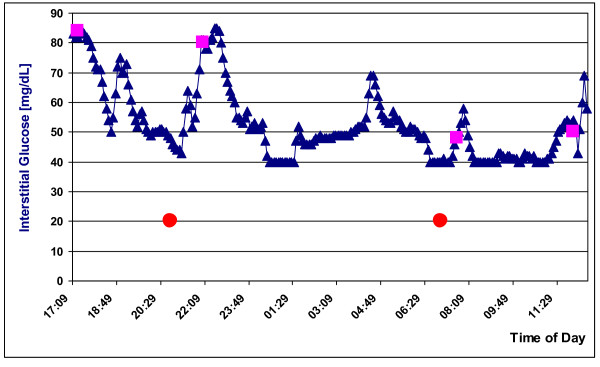
***Real time glucose monitoring *(*RTGM*) in a normoglycemic GMP over ca. 20 h; squares: conventional skin puncture blood glucose measurements; circles: feeding with standard dry feed**.

*RTGM*, a system developed to monitor glucose levels in human diabetics, had been successfully used on GMP by our group [25]. We applied it in two further GMP models. The data in Figure 1 confirms the prior result in one of the two GMP models. The range of values measurable by *RTGM *is 40 mg/dL to 400 mg/dL. Figure 1 shows that interstitial glucose levels in both the fasting (< 70 mg/dL) and postprandial (< 100 mg/dL) non-diabetic control GMP were lower than in non-diabetic humans (range: 80-120 mg/dL). After feeding, interstitial glucose levels in blood rose as expected. In the fasting phase interstitial glucose levels sank temporarily to the lower measurement limit of 40 mg/dL; it can be speculated that the true value was even lower. The four measurements of blood glucose levels obtained manually by skin puncture accorded well with those of the *RTGM*.

#### Intravenous glucose tolerance test in control GMP

**Figure 2:**
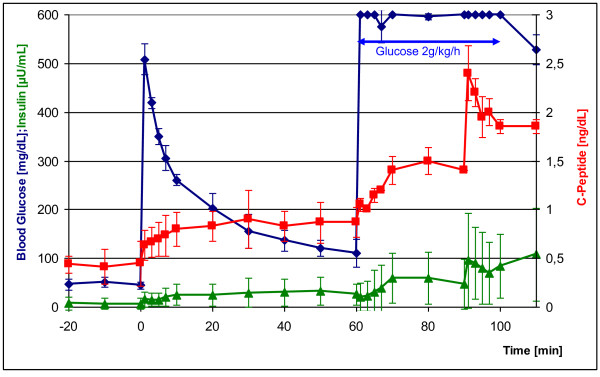
**Intravenous glucose tolerance test (IVGTT) in normoglycemic GMP (n = 3) with subsequent hyperglycemic clamp and arginine injection (blue curve)**. Red curve: C-peptide levels during IVGTT; green curve: insulin levels during IVGTT. Glucose injection at time point t = 0: 0.5 g/kg bw; glucose injection at t = +60 min: 0.6 g/kg; continual glucose infusion from t = +60 min to t = +100 min: 2 g/kg per hour; arginine injection at t = +90 min: 67 mg/kg.

Figure 2 shows blood glucose, insulin and C-peptide levels in serum of the non-diabetic GMP controls before and after intravenous stimulation with glucose. All blood glucose tests were performed exclusively as single measurements. After glucose injection (0.5 g/kg bw) at t = 0 with a starting value of 50 mg/dL, blood glucose levels rose sharply, again attaining almost normoglycemic values with 120 mg/dL at t = +60 min. Within 10 min after glucose injection, C-peptide values increased twofold and remained constant at the higher level. Insulin rose fivefold within 5 min and also remained continuously high. During the ensuing hyperglycemic clamp, continuous application of glucose (initial glucose bolus of 0.6 g/kg followed by glucose infusion of 2 g/kg/h), release of insulin and C-peptide rose again and leveled off at constant values after 10 min. The arginine bolus (67 mg/kg) given at t = +90 min led to peak insulin and/or C-peptide release independent of glucose.

### Glucose metabolism in diabetic STZ-GMP and PE-GMP

Like humans, the GMP were considered to be diabetic if they had a fasting blood glucose level of > 126 mg/dL (7 mmol/L) or a spontaneous blood glucose level of > 200 mg/dL (11.1 mmol/L). C-peptide values after stimulation with glucose did not exceed > 0.4 ng/mL.

#### STZ-induced diabetes

**Figure 3:**
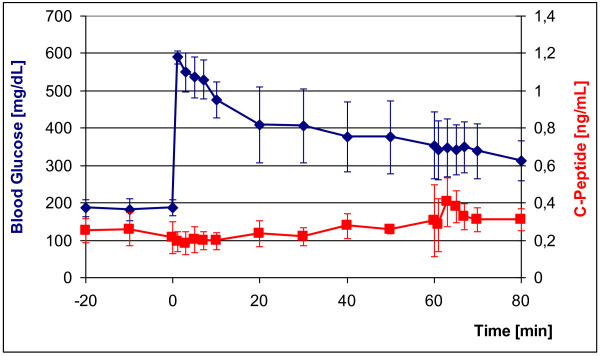
**IVGTT in diabetic GMP (n = 3) 25 days after diabetes induction with STZ (blue curve); red curve: C-peptide concentrations during IVGTT**. Glucose injection at time point t = 0: 0.5 g/kg; arginine injection at time point t = +60 min: 67 mg/kg.

In 5 GMP diabetes was induced with freshly prepared STZ (150 mg/kg body weight). All 5 STZ-GMP became diabetic within 24 h after application of STZ. All STZ-GMP survived STZ application and remained diabetic over the entire observation period of 3 months. Blood glucose-adapted insulin therapy was tested in these animals (see below). Figure 3 shows the IVGTT in 3/5 STZ-GMP. Their blood glucose levels rose rapidly after intravenous glucose application from the diabetic starting levels (180 mg/dL) to 600 mg/dL and declined slowly thereafter as expected; only falling to > 350 mg/dL after 60 min. C-peptide values in serum remained constantly under 0.4 ng/mL over the entire period. With the hyperglycemic clamp (from t = +60 min to t = +80 min) the injection of arginine (67 mg/kg) should have induced release of residual insulin, but blood glucose levels showed no appreciable change, although C-peptide rose temporarily to 0.4 ng/mL.

**Figure 4:**
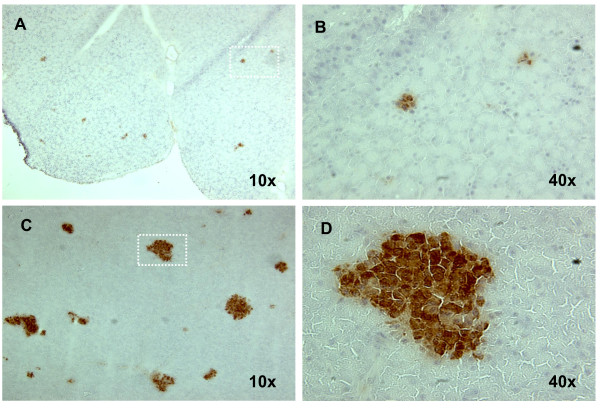
**Residual beta cells in the pancreas of an STZ-GMP**. Pancreas was removed 90 days after STZ-injection; cryosections were stained with an anti-insulin-antibody. (A) Residual beta cells in the pancreas of an STZ-GMP; (B) Detail from A. (C) Beta cells in the pancreas of a normoglycemic GMP (positive control); (D) Detail from C.

At conclusion of the observation time the pancreata of the STZ-GMP were explanted and examined histologically. The pancreata no longer contained intact islets of Langerhans as in the normoglycemic control animals. Residual insulin-positive areas were found at different locations in the sections, possible residue of the once intact islets of Langerhans. Figure 4 shows the results for one of the STZ-GMP.

#### PE-induced diabetes

Diabetes was induced in 5 GMP by total pancreatectomy. All PE-GMP survived the surgical procedure and became diabetic immediately postoperative. Two of the 5 PE-GMP, however, developed an ileus within 4-6 days postoperative and were removed from the study. Learning from this experience, we developed a more intensive postoperative care protocol for the remaining 3/5 PE-GMP: from day +1 to day +14, the remaining 3 PE-GMP were given 1 L sterofundin *per infusionem*, 2 g Novalgin and 20 mg Paspertin daily. Each animal was checked several times daily regarding general health, vitality, blood glucose levels, and gut noises. The following steps were also taken: Insulin and a 40% glucose solution (details below) were given daily, both in dependence on blood glucose levels, potassium (40 mval/d), Neostigmin (0.5 mg/d beginning on day +3 until start of intestinal activity), Tramal (0.7 mg/kg), and the antibiotic Tazobactam 2 × 4.5 g). The PE-GMP also received daily exercise therapy to improve their general health and vitality. A pancreas enzyme (Creon 25.000) was mixed with their dry feed. Under this intensive regimen the PE-GMP began to eat solid feed on their own on the 4th to 7th day postoperative and began to pass stool on the 6th to 8th postoperative day. Within 20 days after total pancreatectomy the PE-GMP exhibited good general health, moved about freely with no complications. In the ensuing time they were given daily exogenous insulin and exogenous pancreas enzyme. Under this protocol the PE-GMP survived without complications until the end of the observation period.

**Figure 5:**
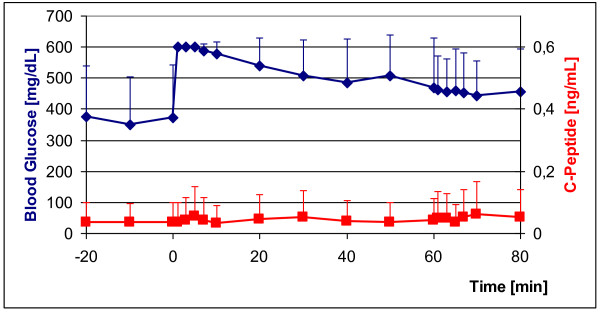
**IVGTT in PE-GMP (n = 3) 25 days after diabetes induction by total pancreatectomy (blue curve); red curve: C-peptide levels during the IVGTT**. Glucose injection at time point t = 0: 0.5 g/kg; arginine injection at t = +60 min: 67 mg/kg.

Figure 5 shows the IVGTT in the 3 PE-GMP. The blood glucose immediately rose from high starting values to > 600 mg/dL, and then fell only slightly over the next 60 min to about 450 mg/dL. C-peptide remained < 0.1 ng/mL at every time point and did not even change after application of glucose and arginine at t = +60 min.

**Figure 6:**
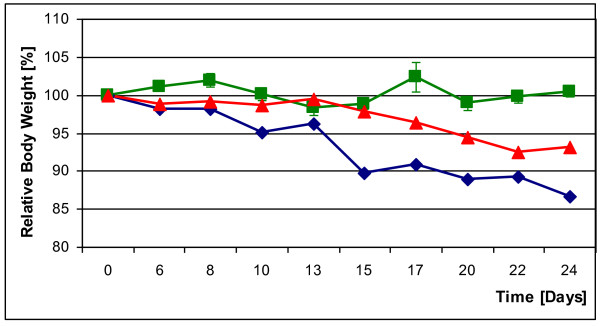
**Body weights of a STZ-GMP (red curve) and a PE-GMP (blue curve) during the first 24 days after diabetes induction; an untreated normoglycemic control GMP (green curve)**.

The weight of the non-diabetic GMP controls remained constant over a period of 24 days, but the diabetic GMP experienced significant weight loss. STZ-GMP lost 6.95% of their starting weight within 24 days; PE-GMP lost twice as much at 13.35%. Figure 6 documents the weight course of all 3 groups over 24 days.

#### Real-time glucose monitoring

**Figure 7:**
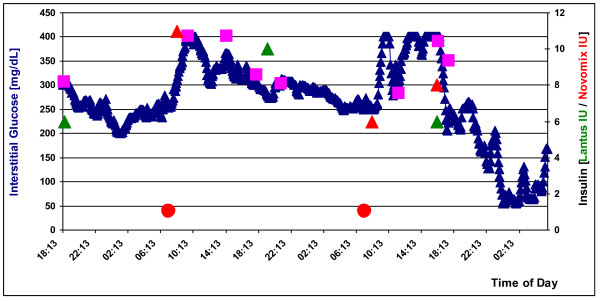
***RTGM *in an STZ-GMP over 60 h**. Squares: conventional skin puncture blood glucose measurements; circle: feedings with standard dry feed; triangles: insulin injections (green: Lantus insulin; red: Novomix insulin).

Independent of the method of diabetes induction, all GMP exhibited a diabetic profile in *RTGM*. Interstitial glucose levels in all GMP met the aforementioned diabetes criteria (Figure 7). After feeding interstitial glucose levels rose sharply and reached the upper limit of the meter at 400 mg/dL, although they may well have been higher. Following application of insulin, interstitial glucose levels fell as expected in proportion to the dose given.

#### Insulin application

In dependence on morning (fasting) blood glucose levels, the GMP were fed dry feed containing insulin (Novomix) consisting of 30% insulin-aspart and 70% intermediary insulin). In the evening (without determining blood glucose levels), they were given the long-acting insulin Lantus, the dose depending on body weight. Following this regimen, the diabetic GMP were maintained without complications at blood glucose levels between 200 and 400 mg/dL over several months. Hypoglycemia was not observed under this protocol.

#### Total costs

The total costs in personnel and time for feeding, housing and caring for the two groups of GMP and to induce diabetes in them are shown in Table 1. STZ-GMP incurred less overall costs than PE-GMP. STZ-GMP were cared for beginning on day +1 by a specially trained animal keeper. In contrast, in the first 14 days postoperative the PE-GMP received daily care from a physician experienced in handling postoperative complications associated with total pancreatectomy. Only after day +14 when the PE-GMP attained stable good general health was their daily medical care taken over by an experience animal keeper.

**Table 1:** Persons, qualifications and time needed to induce the two diabetes models in the Goettingen minipig and care for the diabetic animals prior to grafting the isolated islets of Langerhans.

Type of work	Number of persons and qualification	Work time
diabetes induction with STZ	1 scientist or surgeon 1 animal caretaker	3 hours

diabetes induction with PE	2 surgeons 1 medical assistant 2 anaesthesiologists	6 hours

management of the STZ diabetes	1 animal caretaker	2 × 30 minutes/day

management of the PE diabetes (early phase)	1 surgeon	Up to 4 hours/day

management of the PE diabetes (late phase)	1 animal caretaker	2 × 30 minutes/day

Abbreviations: **STZ: **streptozotocin; **PE: **pancreas explantation in the animal operation theatre (surgical removal of the organ).

## Discussion

The present study shows that both the STZ-GMP and PE-GMP models of diabetes induction produced diabetic GMP. The *RTGM *and IVGTT test procedures reliably measured the diabetic metabolism of the GMP.

### Diabetes induction

All GMP treated with STZ became diabetic within 24 hours; this confirms the data in the literature regarding diabetes induction in GMP using STZ [20]. With regard to diabetes induction using total pancreatectomy and the contradictory data in the literature the following can be said: a) none of our PE-GMP would have survived the procedure without intensive postoperative care, and b) only the intensive drug regimen including insulin application ensured the long-term survival of the PE-GMP - in our animals for 28 days. Due to the total absence of endogenous insulin the application of exogenous insulin seems to be a critical point to ensure long term survival in PE-GMP when compared to the literature [22]. In our experience both diabetes-induction methods are suited for long-term studies. Before beginning such a study researchers should intensively discuss the pros and cons of each model: The PE-GMP model ensures that no endogenous insulin or C-peptide will be produced since the cells that release them are surgically removed, a very important advantage for evaluation of the usually still marginal transplant function. In discussing the drawbacks of the PE-GMP model, a distinction must be made between the *surgical procedure, postoperative care *and *physiology*. Regarding the surgical procedure, it entails a great expenditure in time, technical resources and personnel, and is therefore very expensive. It requires a surgical team experienced in total pancreatectomy. The postoperative care is time intensive and involves tasks that must be performed several times daily until the animal has fully recovered from surgery. The STZ-GMP model, in contrast, can be produced relatively easily at low cost and the critical phase of intensive medical care rapidly gives way to the phase of routine care.

Physiologically the main scientific advantage of the PE-GMP model, namely the complete elimination of host insulin production, also entails considerable drawbacks: Here again a distinction must be made between surgery-related problems and physiological factors. Regarding the former the following can be said: The massive surgical intervention severely compromises the homeostasis of the organism so that the diabetic GMP has major problems with wound healing and is slow to recuperate other bodily functions. Physiologically disadvantageous are the lack of both the anti-insulin hormone glucagon and the exocrine pancreas enzymes essential for digestion. STZ-GMP offer the advantage of ease of diabetes induction (application of central venous catheter) and no prolonged postoperative recuperation phase. Injection of STZ selectively destroys beta cells, leaving the glucagon producing alpha cells intact and thus lessening the risk of hypoglycemia. The exocrine pancreas function also remains intact, and feeding and digestion proceed without complications. The drawback of the STZ-GMP model is the incomplete destruction of beta cells, as confirmed in the present study by the histological detection of residual beta cells and the STZ-GMPs' partial response to a glucose and arginine bolus in IVGTT. For transplantation of islets of Langerhans, therefore, especially if there is inadequate transplant volume, the PE-GMP model is the superior recipient model. For research in the field of metabolic surgery (such as gastric bypass) the STZ-GMS model is better. The goals of metabolic surgery for improvement of glucose homeostasis are to change the incretin *glucagon-like peptide 1 *and *glucose-dependent insulinotropic polypeptide *so as to slow the passage of food and to improve insulin release and/or stimulate the generation of beta cells. These goals would be thwarted by prior abdominal surgery for removal of the pancreas.

### Weight

The continual weight loss in diabetic GMP can be explained by impaired glucose tolerance and is also seen in type 1 diabetics. The clearly greater weight loss in PE-GMP can be attributed to the severity of the surgical intervention and the postoperative drop in food consumption. The control GMP did not gain weight during the observation period, which was expected since they were fully grown adults.

### Intravenous glucose tolerance test

The IVGTT we used with subsequent hyperglycemic clamp is a generally accepted procedure for verification of diabetic disorders [26]. We too could show that this test for monitoring blood glucose, C-peptide and insulin levels can reliably identify diabetes in GMP. The various data however must always be regarded in relation to one another. A simple rise in glucose tolerance above the diabetic threshold is not diagnostic. Independent of C-peptide levels, residual function of the endocrine pancreas may be at play. Unlike blood glucose and C-peptide values, insulin levels in the diabetic GMP lay below the measurement threshold of the methods we used (ELISA). In non-diabetic GMP, by contrast, insulin showed a course comparable to that of C-peptide in IVGTT.

### Real-time glucose monitoring

The *RTGM *we used is a system developed for the diagnosis and control of diabetes. To our knowledge, its use in swine has only been reported by us [25]; the present data confirm and add to our previous results. Once again only measurements are used where the correlation of interstitial glucose and serum glucose levels was > 90%. The data obtained with *RTGM *permit two conclusions: 1) RTGM can be successfully applied preclinically in *swine *as shown in the correlation between our RTGM measurements and the event -- feeding or insulin injection -- influencing glucose homeostasis. 2) RTGM measurements mirror the metabolic state and thus provided valuable evidence regarding the organism's current glucose tolerance. An improvement in glucose tolerance with a slight increase in blood glucose levels after feeding, for example, or a rapid postprandial decline in blood glucose levels are made readily apparent over the long-term.

RTGM's ability to measure long-term trends is a basic difference to IVGTT, which allows only relatively brief monitoring for 120 min after glucose bolus. The major drawback to *RTGM *in swine though is its limited measurement range of 40-400 mg/dL. This range is ideal for human diabetics, since an interstitial glucose level below 40 mg/dL is accompanied by somnolence, if not coma. By nature GMP have lower blood glucose levels than humans, levels which cannot be detected by the device we employed. For future GMP studies a modified *RTGM *system is urgently needed, one that accommodates the greater natural range of swine.

## Conclusion

Both the STZ-GMP and PE-GMP models reliably induce diabetes in GMP and are thus suited for research into specific questions regarding type 1 and type 2 diabetes and/or for preclinical evaluation. To our knowledge we are the first to show that with appropriate postoperative care the PE-GMP can be kept without complications for prolonged periods (week to months), during which, for example, preparations for successful transplantation, e.g. the isolation and *in vitro *culture of several islets of Langerhans transplants, can be made.

## Competing interests

The authors declare that they have no competing interests.

## Authors' contributions

AS designed and performed experiments, analyzed results and wrote the manuscript; VM designed and performed experiments; CT, IC and VW performed experiments; ST and CTG provided critical revision of the manuscript; KU designed experiments, analyzed results and wrote the manuscript. All authors read and approved the final manuscript.
